# Supplementary material for: Discovery and Prediction Study of the Dominant Pharmacological Action Organ of Aconitum carmichaeli Debeaux Using Multiple Bioinformatic Analyses
Source: Int J Mol Sci. 2024 Sep 23;25(18):10219. doi: 10.3390/ijms251810219 (PMC11432385; doi:10.3390/ijms251810219)

Supplementary Table S9. Quantitative analysis of chemical components in *Aconitum carmichaeli* Debeaux extract

Compound name	Molecular Formula	RT (min)	Linear range (ng/mL)	Regression equation	R^2	Content (mg/g)
benzoylmesaconine	$C_{31}H_{43}NO_{10}$	2.85	250-5000	y=11.99978x+1898.13	0.99148	3.36
benzoylaconitine	$C_{32}H_{45}NO_{10}$	3.24	250-5000	y=6.08927x+1241.752	0.99131	0.84
aconitine	$C_{34}H_{47}NO_{11}$	4.90	250-5000	y=12.72896x+1933.095	0.99427	ND

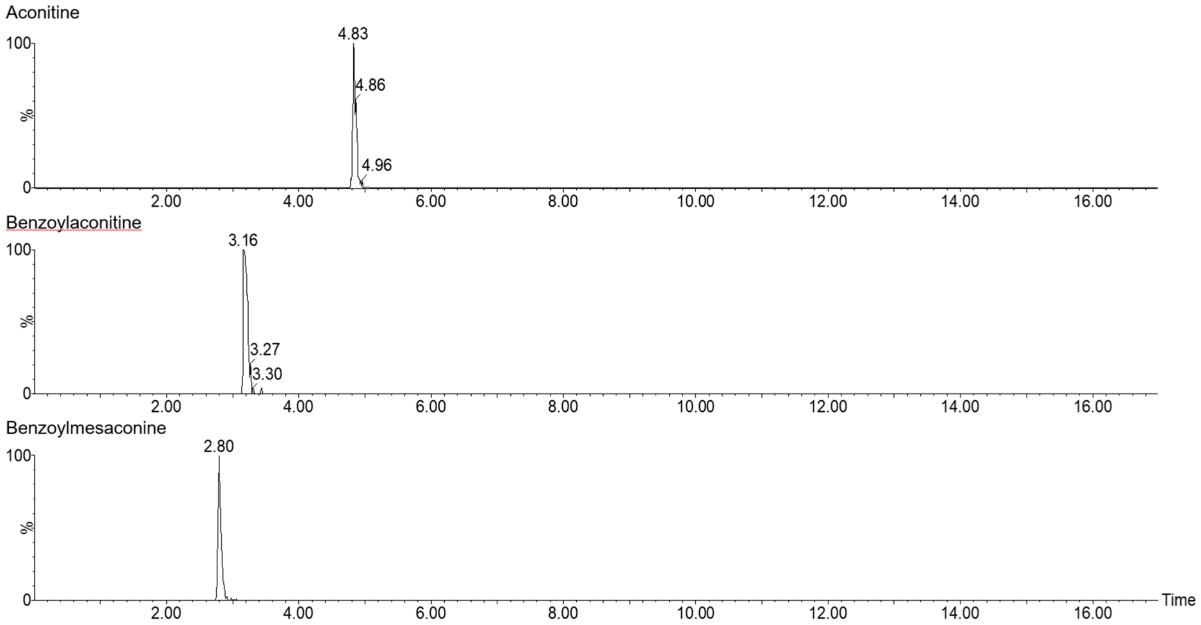

Supplement: Supplementary file 1 [file ijms-25-10219-s001.zip › Supplementary table S9_Quantitative analysis of chemical components in Aconitum carmichaeli Debeaux extract.pdf]
