# Supplementary material for: Discovery and Prediction Study of the Dominant Pharmacological Action Organ of Aconitum carmichaeli Debeaux Using Multiple Bioinformatic Analyses
Source: Int J Mol Sci. 2024 Sep 23;25(18):10219. doi: 10.3390/ijms251810219 (PMC11432385; doi:10.3390/ijms251810219)
Supplement: Supplementary file 1 [file ijms-25-10219-s001.zip › Supplementary_figure_legend.pdf]

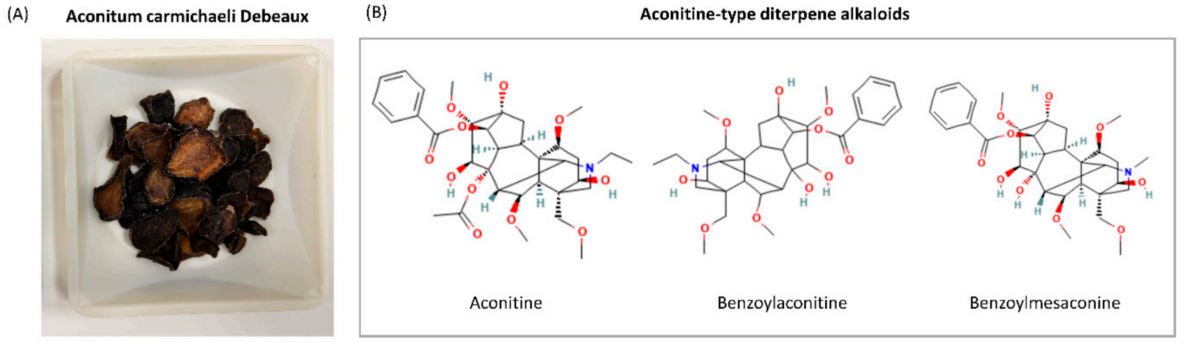

Supplemental Figure S1. ACD images and structures of major aconitine-type diterpene alkaloids. (A) Image of truncated ACD used as a drug. (B) Structural information of three aconitine-type diterpene alkaloids, which are major compounds of ACD. ACD, Aconitum carmichaeli Debeaux

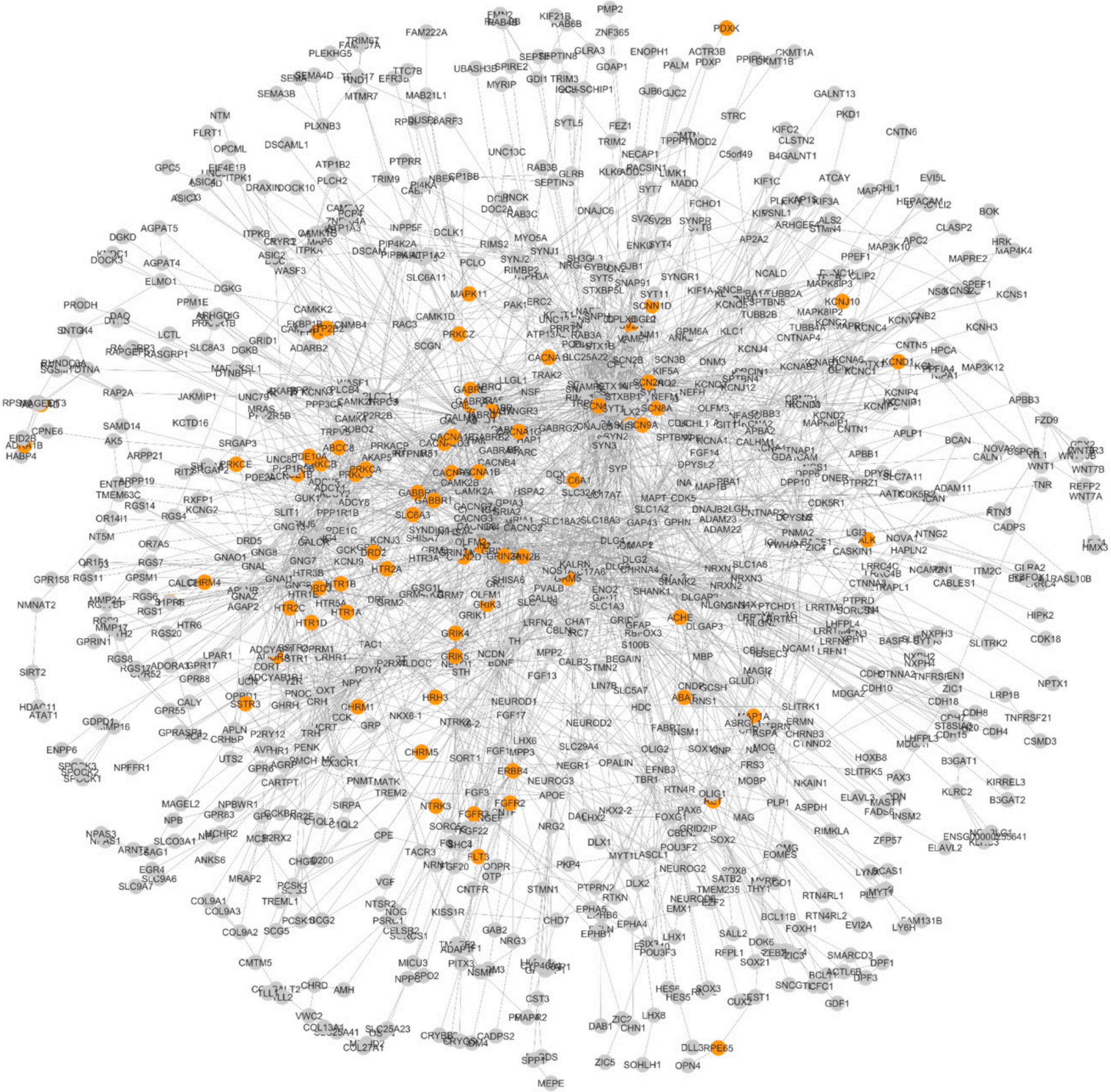

Supplementary Figure S2. Enlarged image of BSN. This image is reprocessed to better show the node information of BSN used in the main text. Orange nodes are dockable proteins that have more than 10 hits in the docking analysis results. BSN, brain system network.

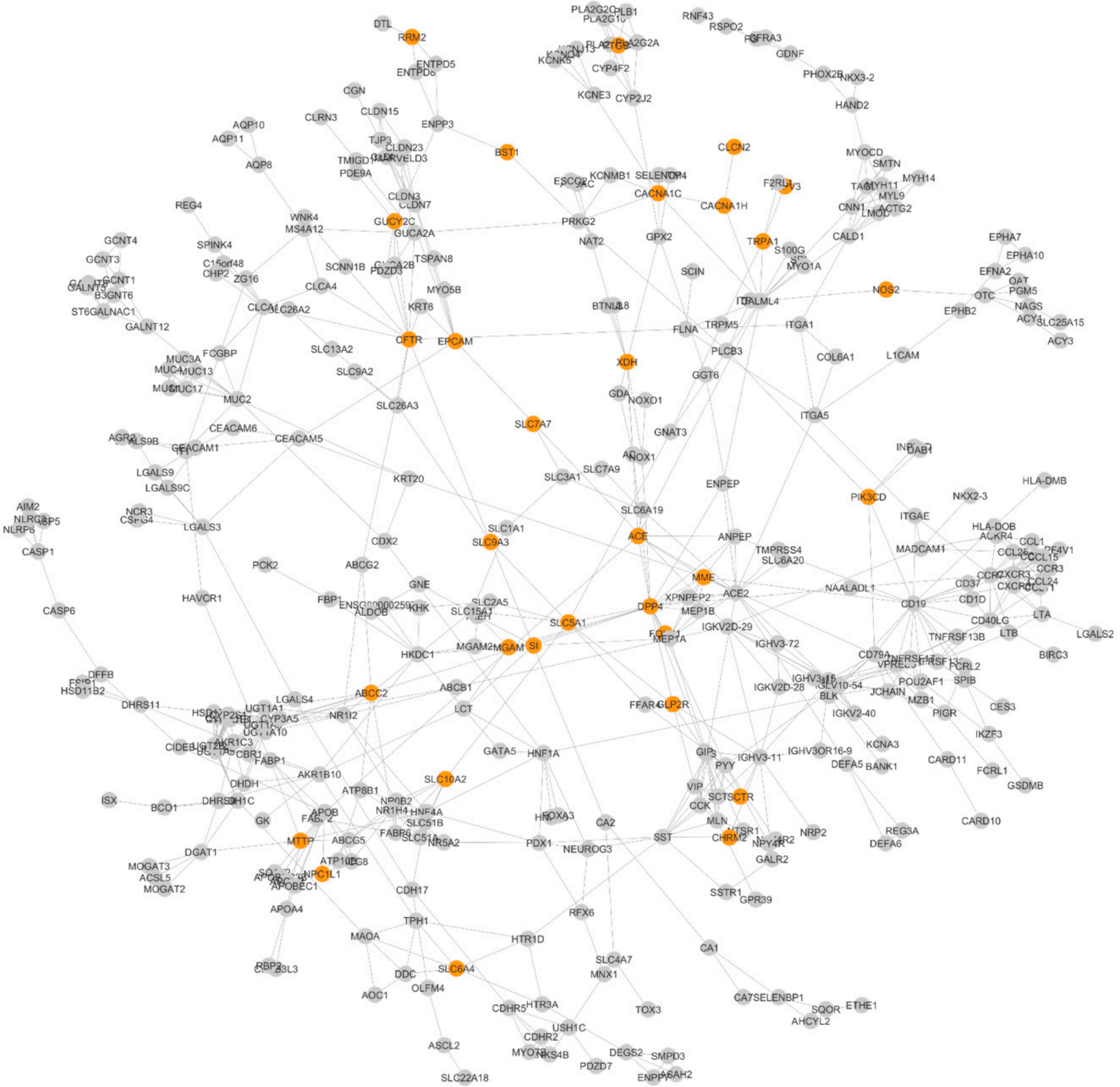

Supplementary Figure S3. Enlarged image of ISN. This image is reprocessed to better show the node information of ISN used in the main text. Orange nodes are dockable proteins that have more than 10 hits in the docking analysis results. ISN, intestinal system network.

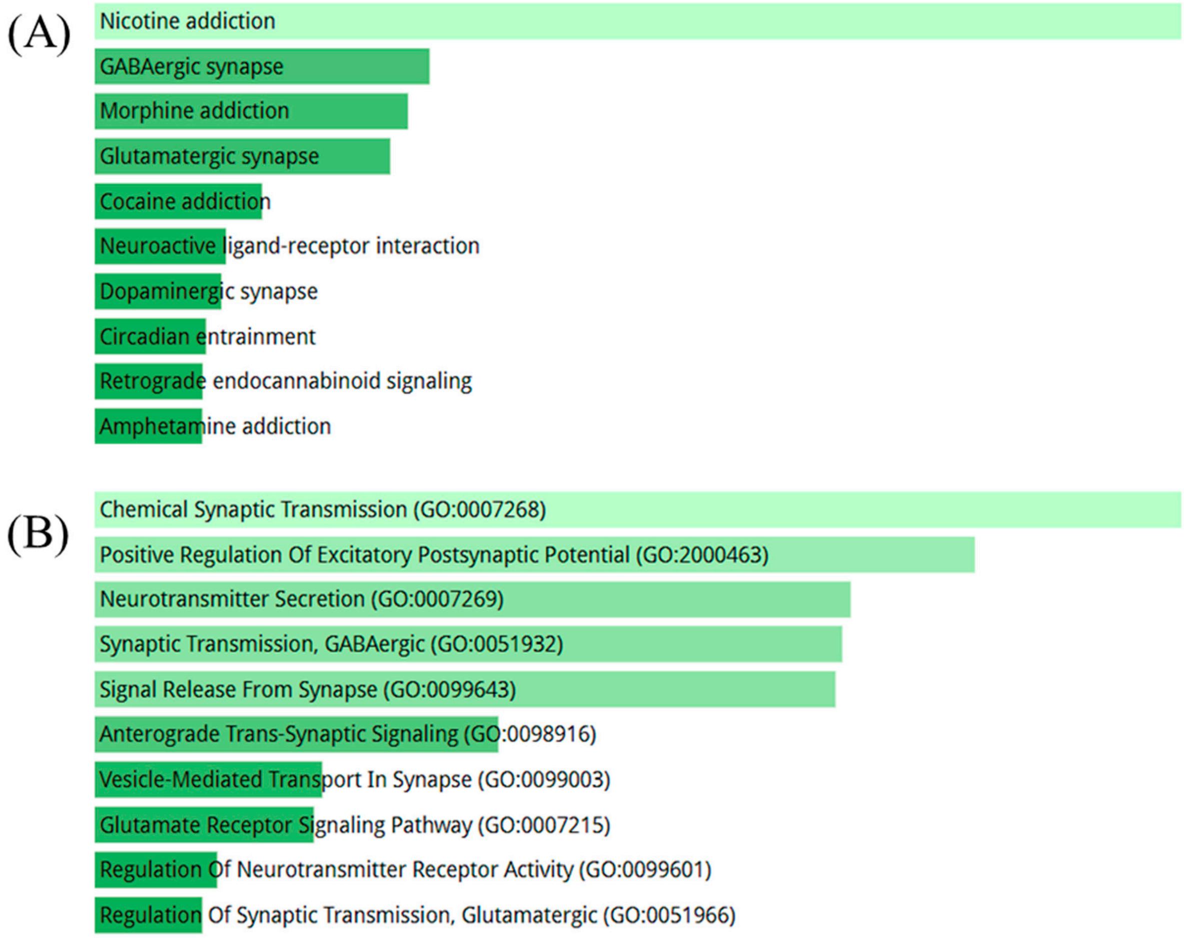

Supplementary Figure S4. ORA results using first-shell interaction of BSN. on the BSN using DP of ACD that act on the brain proteome and ACD-induced DEG of the SW1783 cell line, and neighbor nodes of the two proteins list on the BSN. (A) ORA results using KEGG pathway gene set (B) ORA results using GOBP gene set. ORA, Over-representation analysis; BSN, brain system network; DP, druggable proteomes; ACD, *Aconitum carmichaeli* Debeaux; DEG, differentially expressed genes; KEGG, Kyoto Encyclopedia of Genes and Genomes; GOBP, Gene Ontology biological process

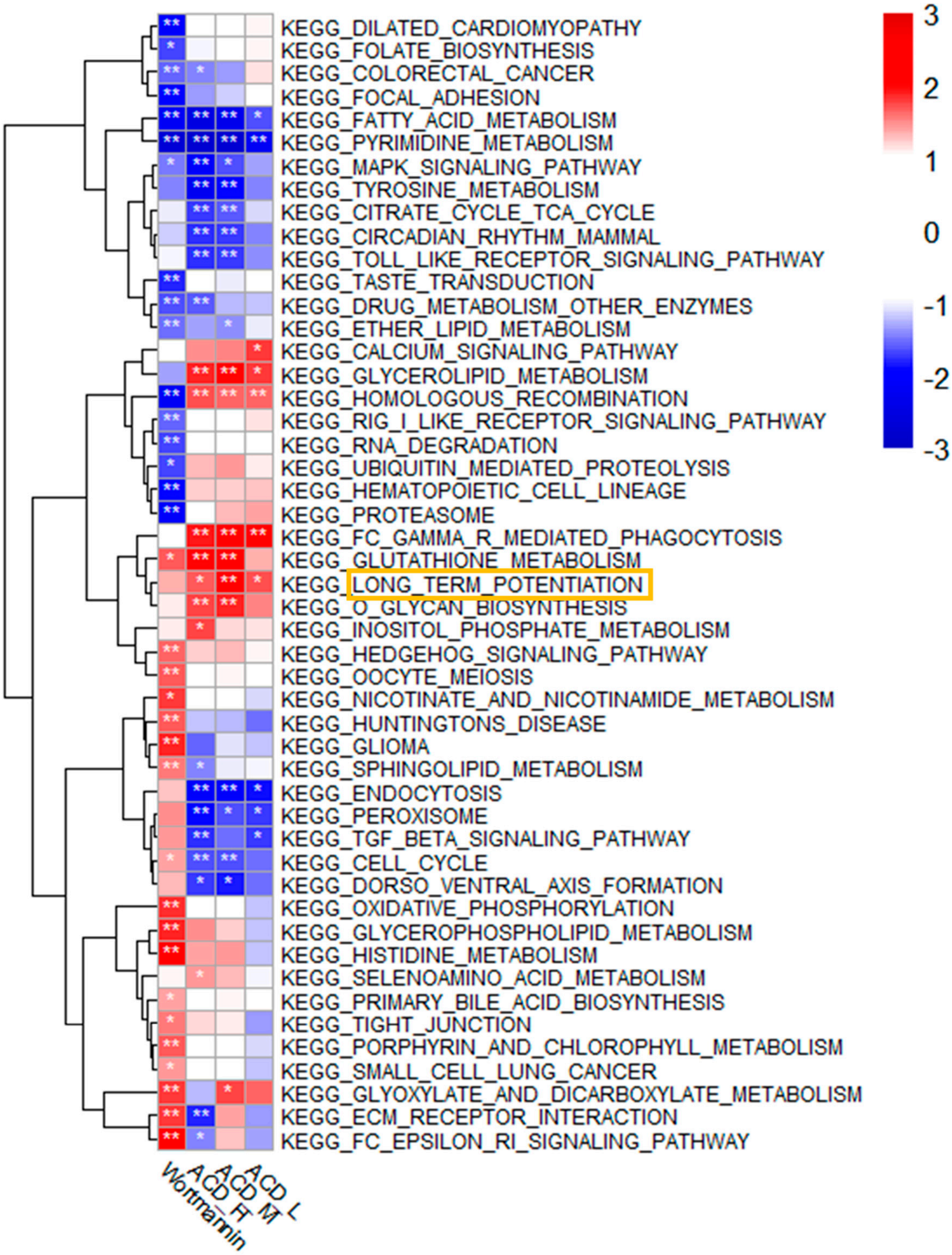

Supplementary Figure S5. GSEA results based on KEGG pathway gene set performed using transcriptome expression results of SW1783 cell line treated with ACD. *, p-value<0.05. **, p-value<0.01. GSEA, Gene set enrichment analysis; KEGG, Kyoto Encyclopedia of Genes and Genomes; ACD, *Aconitum carmichaeli* Debeaux

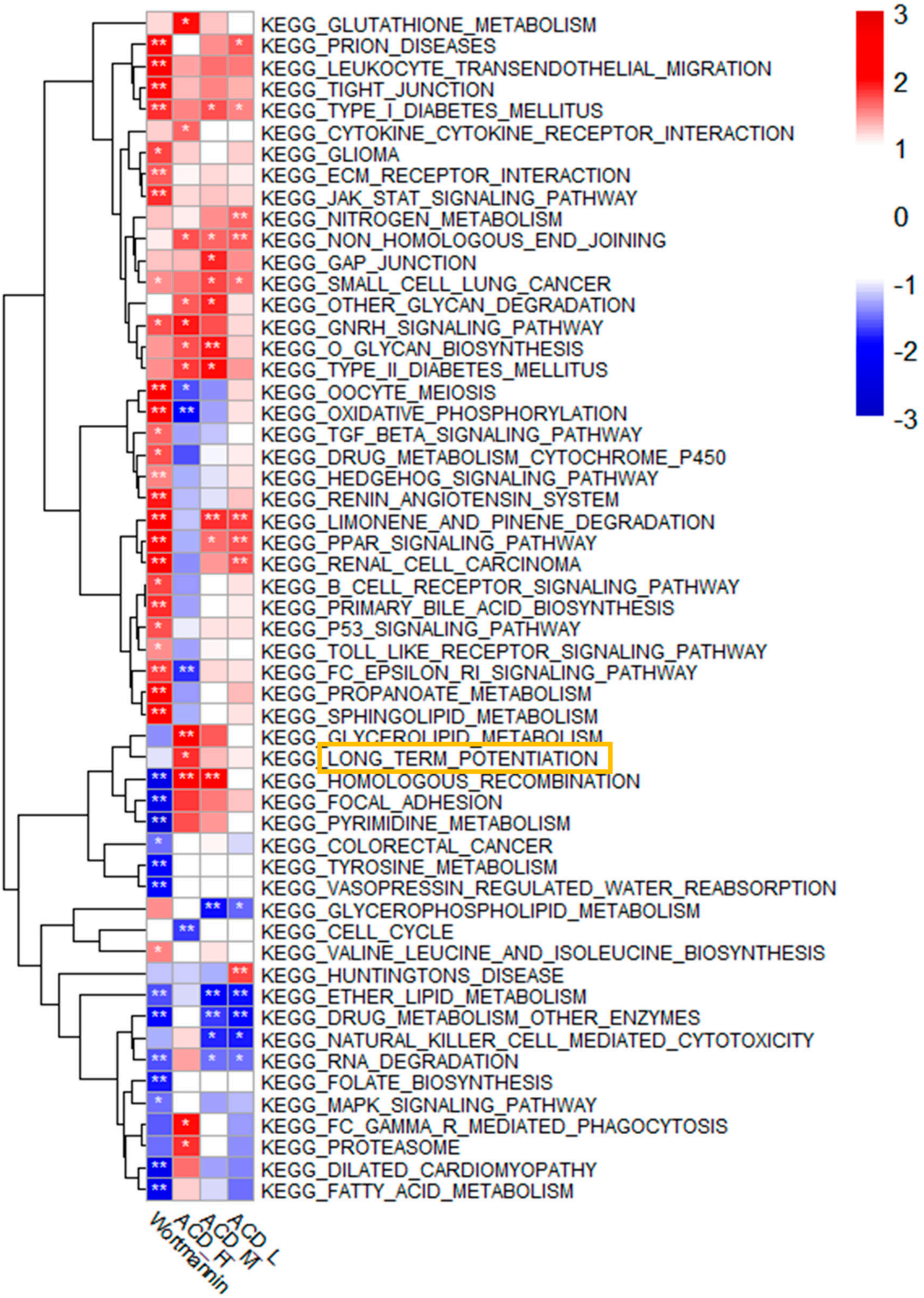

Supplementary Figure S6. GSEA results based on KEGG pathway gene set performed using transcriptome expression results of HT29 cell line treated with ACD. *, p-value<0.05. **, p-value<0.01. GSEA, Gene set enrichment analysis; KEGG, Kyoto Encyclopedia of Genes and Genomes; ACD, *Aconitum carmichaeli* Debeaux

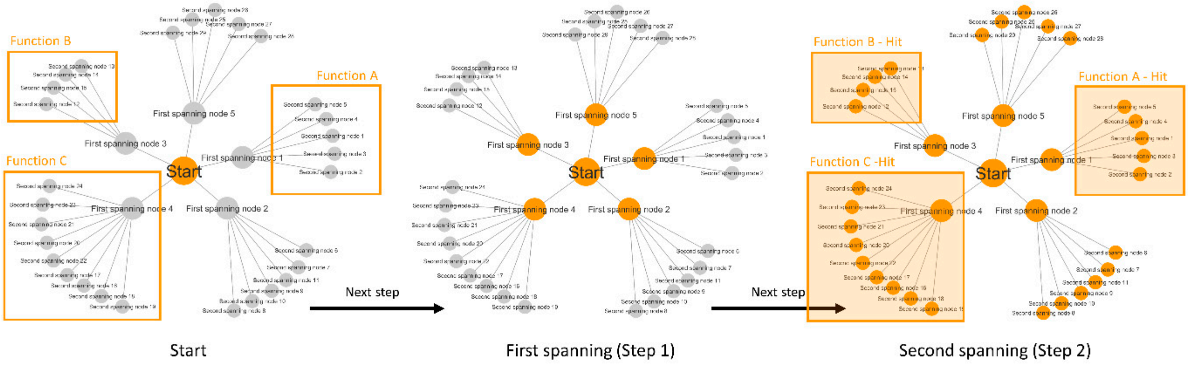

Supplementary Figure S7. Schematic diagram of network spanning. This is a schematic diagram explaining network spanning. Proteins interact with other proteins in the human body, and it calculates how many steps are needed to span the entire network. It is suggested that a specific network composed of specific proteins and proteins with different roles has dimensions as many as the roles, and that the functions of the complete organ are activated only when all dimensions that play a role are activated.
