# Supplementary figures and images for: Discovery and Prediction Study of the Dominant Pharmacological Action Organ of Aconitum carmichaeli Debeaux Using Multiple Bioinformatic Analyses
Source: Int J Mol Sci. 2024 Sep 23;25(18):10219. doi: 10.3390/ijms251810219 (PMC11432385; doi:10.3390/ijms251810219)

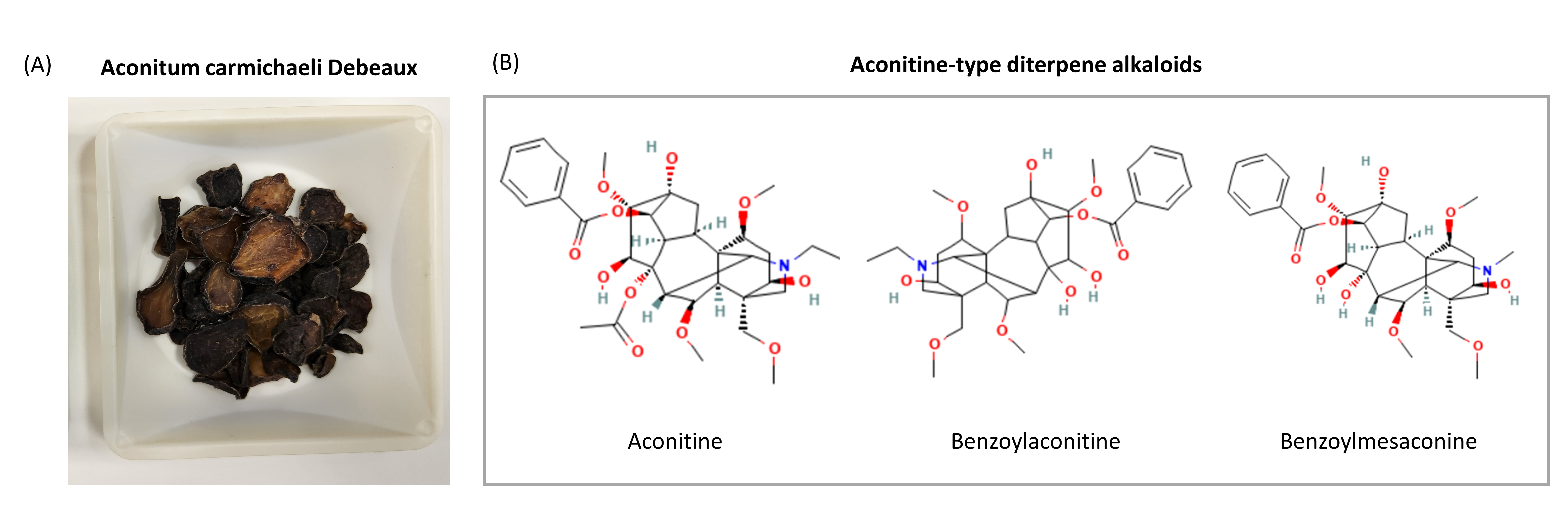

Supplement: Supplementary file 1 [file ijms-25-10219-s001.zip › Supplementary figure S1.tif]

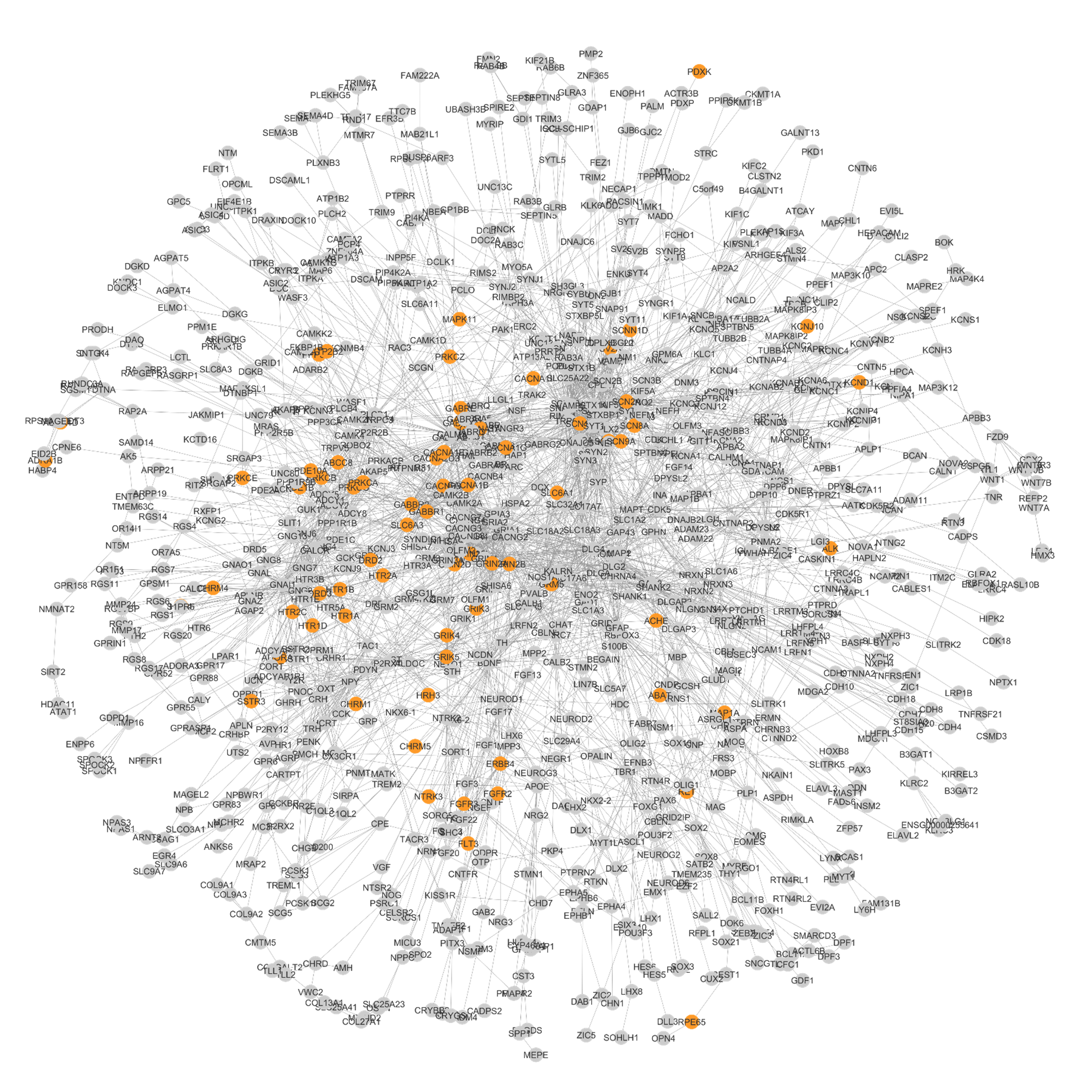

Supplement: Supplementary file 1 [file ijms-25-10219-s001.zip › Supplementary figure S2.tif]

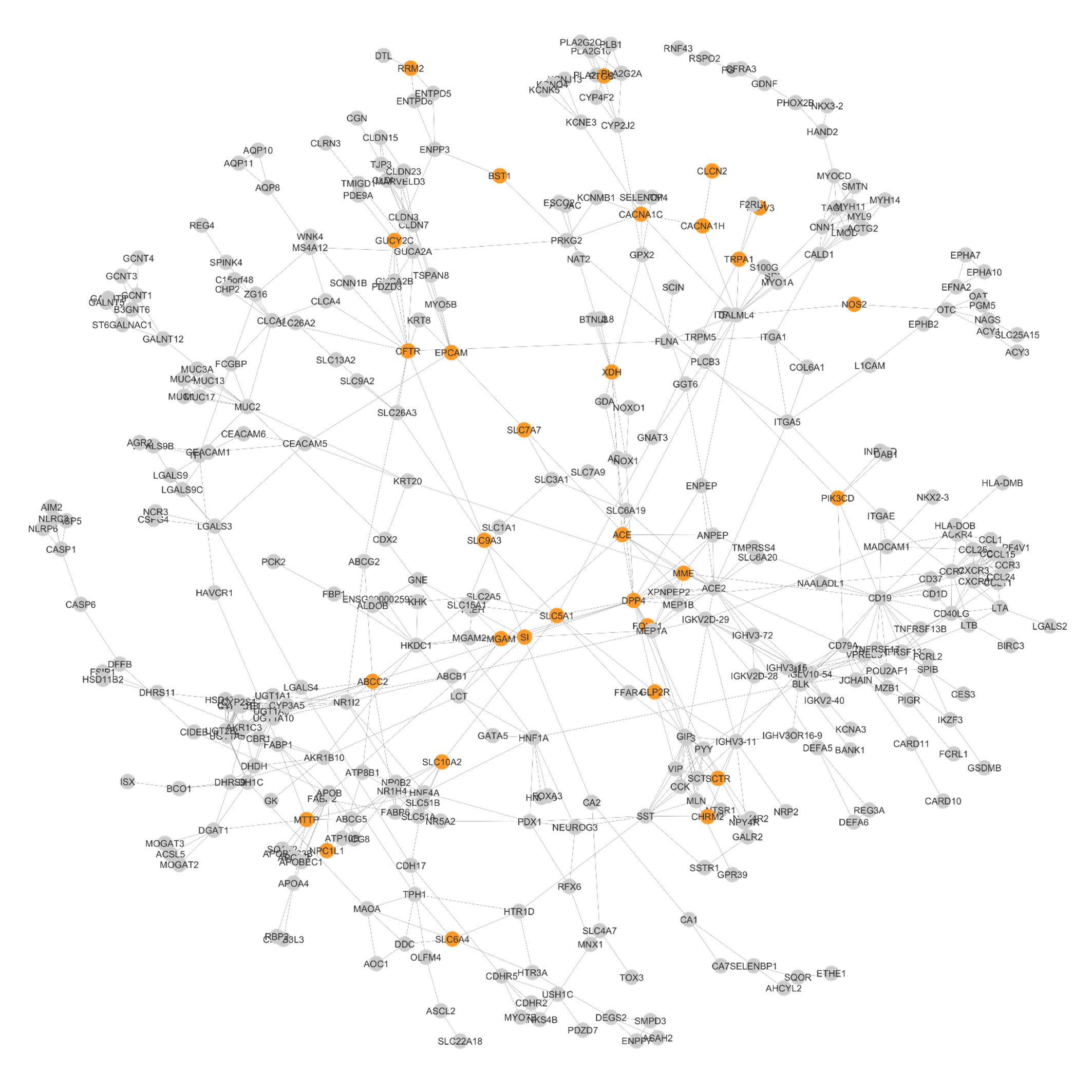

Supplement: Supplementary file 1 [file ijms-25-10219-s001.zip › Supplementary figure S3.tif]

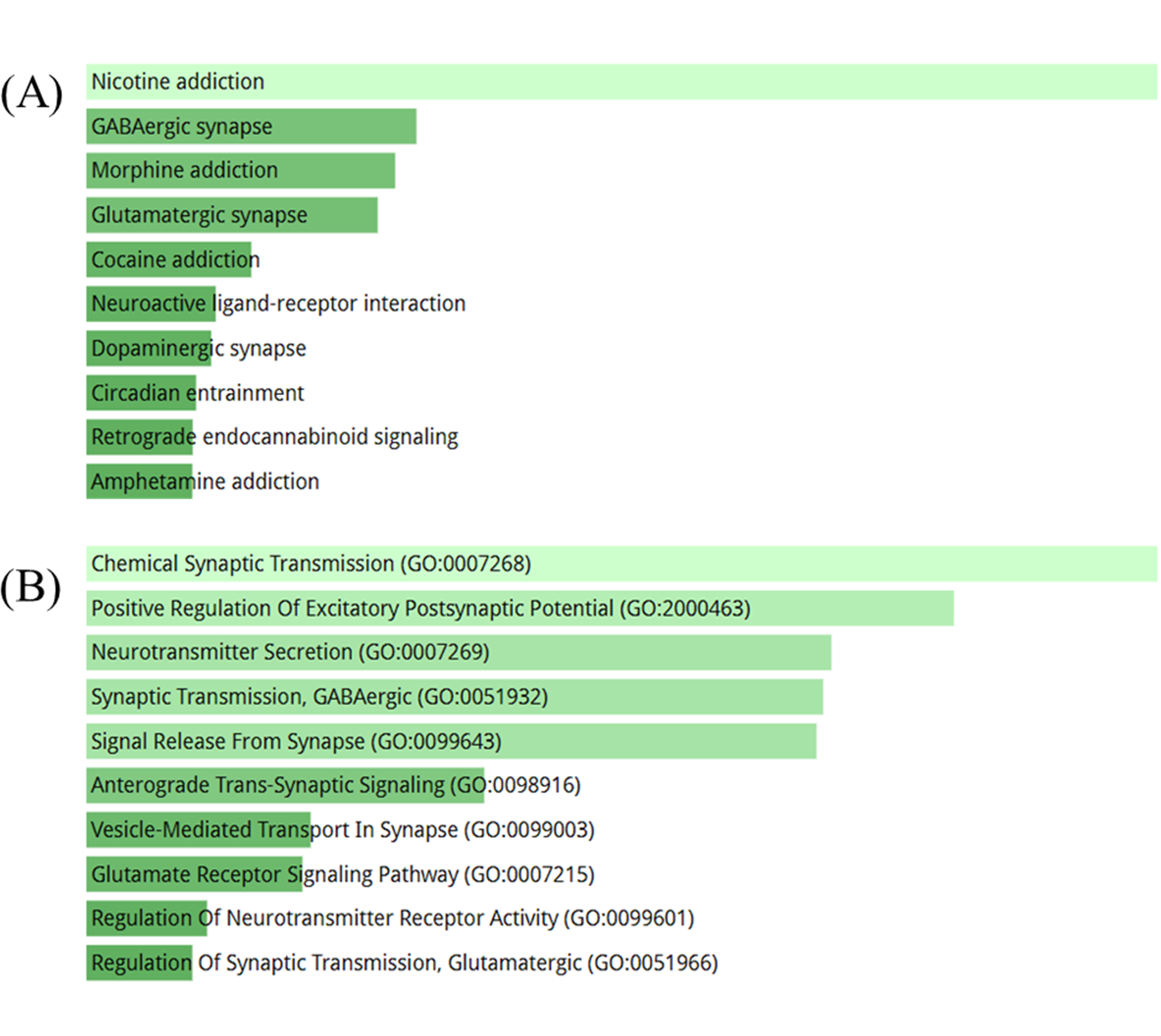

Supplement: Supplementary file 1 [file ijms-25-10219-s001.zip › supplementary figure S4.tif]

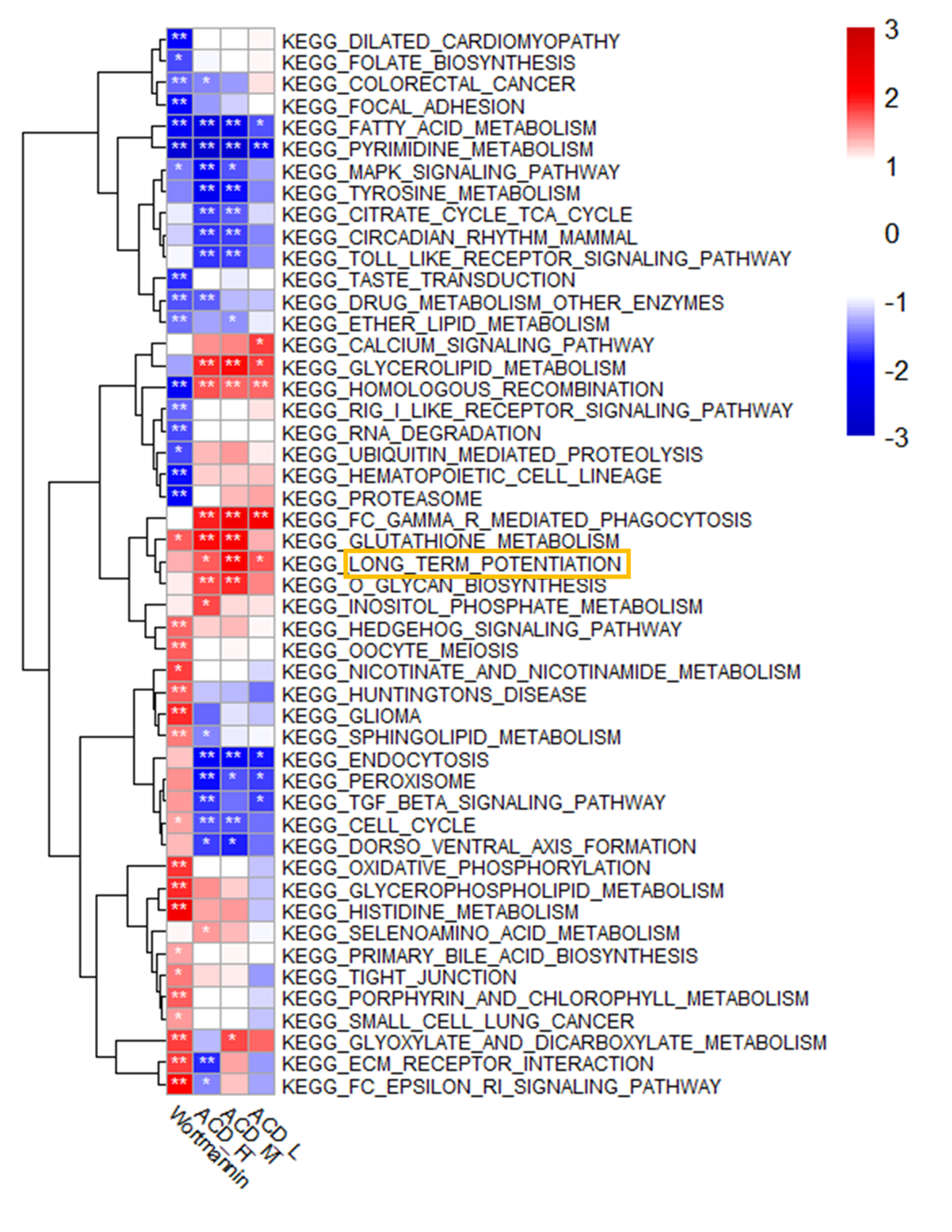

Supplement: Supplementary file 1 [file ijms-25-10219-s001.zip › supplementary figure S5.tif]

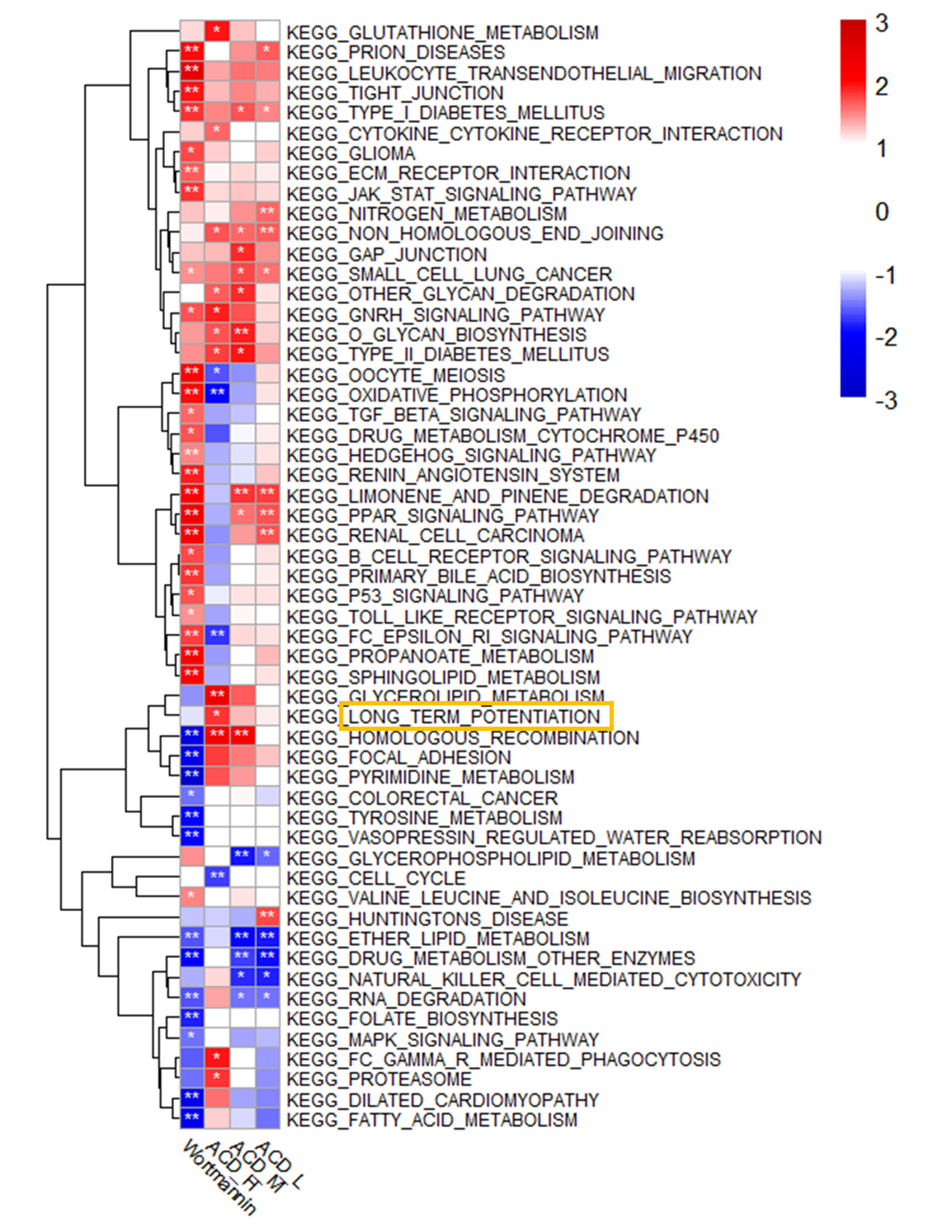

Supplement: Supplementary file 1 [file ijms-25-10219-s001.zip › supplementary figure S6.tif]

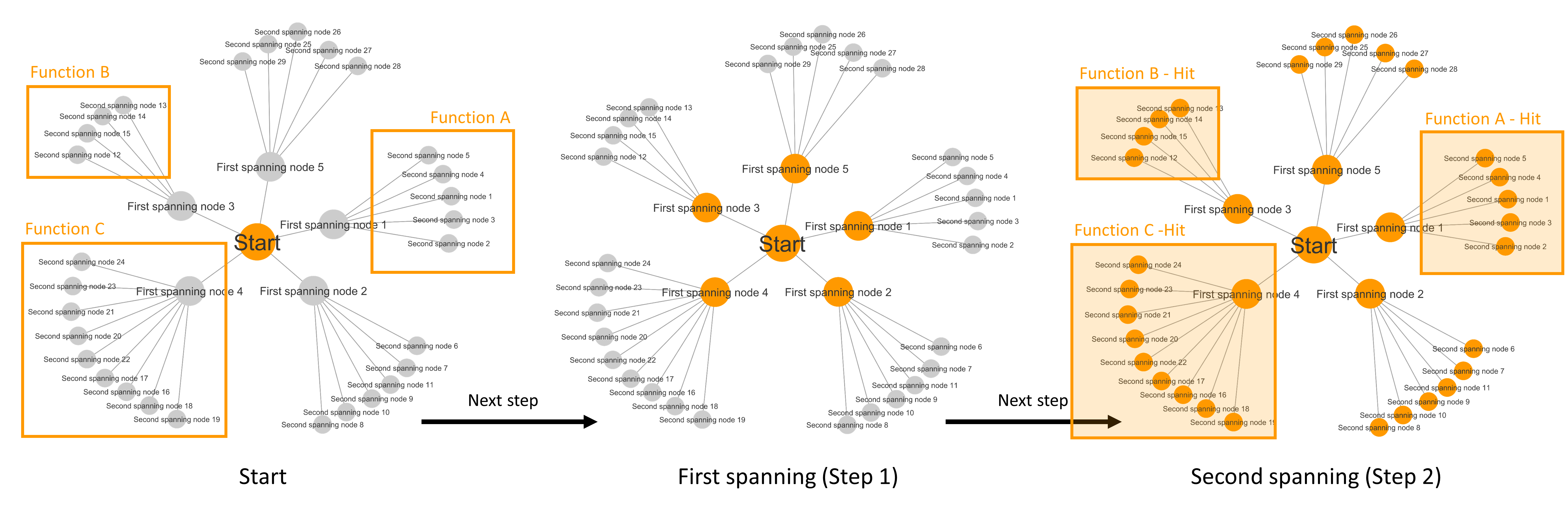

Supplement: Supplementary file 1 [file ijms-25-10219-s001.zip › Supplementary figure S7.tif]
